# Improved Detection of *in vivo* Human NK Cell-Mediated Antibody-Dependent Cellular Cytotoxicity Using a Novel NOG-FcγR-Deficient Human IL-15 Transgenic Mouse

**DOI:** 10.3389/fimmu.2020.532684

**Published:** 2020-10-07

**Authors:** Ikumi Katano, Ryoji Ito, Kenji Kawai, Takeshi Takahashi

**Affiliations:** ^1^Laboratory Animal Research Department, Central Institute for Experimental Animals (CIEA), Kawasaki, Japan; ^2^Pathological Analysis Center, Central Institute for Experimental Animals (CIEA), Kawasaki, Japan

**Keywords:** humanized mice, NK cell, FcγR, ADCC, mouse innate immunity

## Abstract

We generated an NOD/Shi-*scid*-IL2Rγ^*null*^ (NOG) mouse deficient for the *Fcer1g* and *Fcgr2b* genes (NOG-FcγR^−/−^ mice), in which monocytes/macrophages do not express activating (FcγRI, III, and IV) or inhibitory (FcγRIIB) Fcγ receptors. Antibody-dependent cellular cytotoxicity (ADCC) by innate immune cells was strongly reduced in this strain. Thus, while the growth of xenogeneic human tumors engrafted in conventional NOG mice was suppressed by innate cells upon specific antibody treatment, such growth inhibition was abrogated in NOG-FcγR^−/−^ mice. Using this novel strain, we further produced NOG-FcγR^−/−^-mice expressing human IL-15 (NOG-FcγR^−/−^-hIL-15 Tg). The mice inherited unique features from each strain, i.e., the long-term sustenance of human natural killer (NK) cells, and the elimination of mouse innate cell-mediated ADCC. As a result, segregation of human NK cell-mediated ADCC from mouse cell-mediated ADCC was possible in the NOG-FcγR^−/−^-hIL-15 Tg mice. Our results suggest that NOG-FcγR^−/−^-hIL-15 Tg mice are useful for validating the *in vivo* function of antibody drug candidates.

## Introduction

Antibody-dependent cellular cytotoxicity (ADCC) by natural killer (NK) cells has been considered a critical mechanism in antibody therapy for various malignant diseases, including hematopoietic malignancies and some solid tumors (1). Advances in technology have dramatically accelerated the development of various types of novel antibody (2–4), whereas there are few *in vivo* animal models to use to validate candidates in terms of pharmacokinetics (5) and pharmacodynamics, as they are directed against human antigens and are often developed in the form of humanized or even human IgG molecules. The absence of target antigens in conventional mouse models has limited the progress of drug discovery. Xenograft models in immune-deficient mice or genetically manipulated mice, which express human antigens (6) as surrogate targets, have been employed as *in vivo* models. However, they do not always assure faithful extrapolation to human situations, because such models lack human effectors cells, including NK cells or T cells. Thus, innovative experimental animal models are sought to enable the accurate predictions of drug efficacy and effects on human cells and tissues.

Humanized mice, which stably and autonomously maintain human tissues, have attracted significant attention (7–9). Extremely immunodeficient mice, such as NOD/Shi-*scid*-IL2Rγ^*null*^ (NOG) (10), NOD/LtSz-scid IL-2Rγ^null^ (NSG) (11), or BALB-RAG2^−/−^-IL-2Rγ^−/−^ double knockout (BRG) mice (12) or the derivative strain, BRG-human signal-regulatory protein α (SIRPA) (BRGS) (13), are among the best platforms for the reconstitution of human hematopoietic and immune systems, by transplantation of human hematopoietic stem cells (HSCs) or human peripheral blood mononuclear cells (PBMCs). Application of these mice in the evaluation of ADCC has also been attempted (14–16). Our group previously reported that two NOG variant strains, that express either human IL-2 (hIL-2) or human IL-15 (hIL-15); NOG-hIL-2 transgenic (Tg) or NOG-hIL-15 Tg, respectively, are suitable for human NK cell development (17) and/or long-term persistence of human NK cells (18). These human NK cells suppress human tumor growth *in vivo* by cognate or therapeutic antibody-dependent mechanisms (17, 18). In the course of studies using various therapeutic and research antibodies, however, we have realized that tumor growth is significantly suppressed by antibody treatment alone in some cases, even in the absence of human NK cells. Thus, the capability of antibodies to induce human NK-cell mediated ADCC is often masked by this background effect.

In this study, we established an NOG-FcγR deficient (NOG-FcγR^−/−^) strain. While administered therapeutic antibody in NOG mice was mainly captured by innate cells, primarily neutrophils and monocytes/macrophages, this did not occur in NOG-FcγR^−/−^ mice. Accordingly, innate cell-mediated ADCC was strongly suppressed in NOG-FcγR^−/−^ mice. Furthermore, we generated an NOG-FcγR^−/−^-hIL-15 Tg mouse, and demonstrated that specific detection of human NK cell-mediated ADCC was possible, suppressing interference from mouse innate cells.

## Materials and Methods

### Mice

NOG, NOG-hIL-15 Tg [formally NOD.Cg-*Prkdc*^*scid*^*il2rg*^*tm*1*Sug*^ *Tg (CMV-IL15)1 Jic*/Jic] (18), and NOG-FcγR^−/−^ mice were used in this study. These strains were maintained in the Central Institute for Experimental Animals (CIEA) under specific pathogen-free conditions. To generate NOG-FcγR^−/−^ mice, frozen embryos of NOD.Cg-Fcer1g^ < tm1Rav>^ Fcgr2b^ < tm1Ttk>^ were obtained from RIKEN BRC (RBRC02330, Tsukuba, Japan) (19). The mice were backcrossed with NOG mice to introduce the *scid* mutation and the IL-2Rγc targeted allele (20). NOG-hIL-15 Tg and NOG-FcγR^−/−^ mice were crossed to produce NOG-FcγR^−/−^-hIL-15 Tg mice. All experiments were performed in accordance with institutional guidelines (14038, 17025, 20044), which were approved by the Animal Experimentation Committee of CIEA.

### Reagents

Antibodies for flow cytometric analyses were as follows.

For staining mouse cells, anti-FcεRIα-fluorescent isothiocyanate (FITC; clone, Mar-1), anti-CD64 (FcγRI)-Phycoerythrin (PE; X54-5/7.1), anti-F4/80-PE (BM8), anti-CD16/32 (FcγRIII/II)-PE/Cyanine 7 (Cy7; 93), anti-Gr-1-Allophycocyanin (APC; RB6-8C5), and anti-CD45- APC/Cy7 (30-F11) were from Biolegend (San Diego, CA). For human NK cell staining, anti-CD45-Brilliant Violet (BV) 510 (HI30), anti-CD3-BV421 (UCHT1), anti-CD56-AlexaFluor700 (HCD56), anti-CD56-PE/Cy7 (HCD56), anti-CD16-BV605 (3G8), and anti-CD335 (NKp46)-PE (9E2) were from Biolegend. Anti-CD19-BUV395 (SJ25C1), anti-CD14-APC/Cy7 (HCD14), and anti-CD314 (NKG2D)-APC (1D11) were from BD Biosciences (San Jose, CA). For detecting human IgG captured by mouse cells, anti-human IgG-FITC (G18-145, BD Biosciences) was used.

Anti-HER2 antibody (Trastuzumab, Herceptin®) and anti-CD20 antibody (Rituximab, Rituxan®) were purchased from Chugai (Tokyo, Japan). Anti-CCR4 antibody (Mogamulizumab, Poteligeo®) was from Kyowa-Kirin (Tokyo, Japan).

### Flow Cytometry

Spleen cells were prepared by smashing the tissues between two glass slides and filtration over nylon mesh. Red blood cells (RBCs) were lysed with RBC lysing solution (Pharm Lyse; BD Biosciences). After washing, the cell pellet was resuspended in PBS containing 0.1% bovine serum albumin (BSA). Bone marrow (BM) cells were recovered by flushing femurs with PBS + 0.5% BSA. After debris removal over nylon mesh and centrifugation, the pellets were resuspended in 1 mL PBS + 0.1% BSA. Thus, prepared single mononuclear cell suspensions were stained with the indicated antibodies in fluorescence-activated cell sorting (FACS) buffer (PBS containng 0.1% BSA and 0.05% NaN_3_) for 30 min at 4°C in the dark. After washing in FACS buffer, cells were resuspended in FACS buffer containing propidium iodide (PI) and subsequently subjected to multicolor flow cytometric analyses on a FACSCanto or Fortessa instrument (BD Biosciences). The data were analyzed using FACSDiva software (ver. 6.0.4 or 8.0.3; BD Biosciences) or FlowJo^TM^ (ver10; BD Biosciences).

### Quantification of Immunoglobulin by Enzyme-Linked Immunosorbent Assay (ELISA)

The levels of human IgG or mouse IgG in the plasma of mice injected with therapeutic or control antibodies, respectively, were determined using ELISA kits from Bethyl Laboratories (Human IgG or Mouse IgG ELISA Quantitation Sets, Montgomery, TX) according to the manufacturer's protocol. Peripheral blood (PB) was collected at the indicated time points from the retro-orbital venous plexus after anesthetization of mice with isoflurane. The plasma was recovered by centrifugation (400 g × 10 min) and subjected to ELISA.

To determine the levels of human IL-15 or mouse IL-15 in mouse plasma, we used a LEGEND MAX^TM^ Human IL-15 ELISA kit (Biolegend) or mouse IL-15 DuoSet® ELISA (R&D systems, Inc., Minneapolis, MN), respectively.

### Cell Culture

Daudi (CD20^+^ Burkitt lymphoma) and 4–1ST (HER-2^+^ gastric cancer) (21, 22) cell lines were used in this study. Daudi cells were cultured in complete RPMI-1640 medium (Gibco, Grand Island, NY, USA) with 10% FCS and antibiotics (penicillin-streptomycin, Gibco). The 4–1ST cells were maintained by serial transplantation in NOG mice, i.e., a 2–3 mm^3^ piece of tumor was excised from an established tumor by razor blade and inoculated into the flank of a new recipient mouse under anesthesia.

Human NK cells were expanded using BINKIT® (Biotherapy Institute of Japan inc., Tokyo, Japan), according to the manufacturer's instructions. Briefly, 25 mL peripheral blood was collected from donors after obtaining informed consent. Total PBMCs were separated over a density gradient, using Lymphoprep^TM^ (Alere Technologies AS, Oslo, Norway) and subsequent washing. We used BINKIT to culture 8.5 × 10^7^ PBMC (containing about 2.5 × 10^7^ NK cells). After 15 days expansion, cultured NK cells were purified by negative sorting, using a human CD56^+^ NK cell isolation kit and magnetic cell separation (MACS, Miltenyi Biotec, Bergisch Gladbach, Germany). The degree of amplification of NK cells was about 18-fold. The expanded cells were frozen in Cell Banker1® (Takara, Shiga, Japan) and cryopreserved in a LN2 tank. For *in vivo* transplantation, thawed NK cells were further purified by eliminating human CD3^+^ T cells (EasySep^TM^ Human CD3 positive selection kit II, STEMCELL^TM^ technologies, Vancouver, Canada). Typical purity after sorting was more than 99%, and the cells included CD56^+^ CD16^+^ NKp46^+^ NKG2D^+^, with few CD3^+^ human T cells (Supplementary Figure 1).

### *In vivo* Tumor Transplantation Model

NOG-FcγR^+/−^ and NOG-FcγR^−/−^ mice were inoculated subcutaneously in the flank with 4–1ST cells as mentioned above. Antibody treatment was begun at 7 days post inoculation. Anti-HER2 antibody (Trastuzumab, 50 μg) was injected i.p. twice per week for 2 weeks. Tumor size was measured every week using a caliper and calculated using the following formula: tumor volume (mm^3^) = 1/2 × length (mm) × [width (mm)]^2^.

For experiments using Daudi cells and anti-CD20 antibody (Rituximab), mice were transplanted with 1.5 × 10^6^ Daudi cells via tail vein (day 0) and injected i.p. with 50 μg Rituximab once per week over 3 weeks. In some experiments, *in vitro* expanded NK cells (5 × 10^6^) were transplanted twice, on days 3 and 10. The engraftment of Daudi cells was monitored every week by flowcytometry of peripheral blood samples.

### Immunohistochemistry and Pathological Examination

Kidneys from Daudi-bearing mice were fixed in 10% neutralized formalin (Mildform, FUJIFILM Wako Pure Chemical, Osaka, Japan). Formalin-fixed tissues were embedded in paraffin and analyzed by either hematoxylin-eosin staining (H&E) or immunohistochemistry. Staining of sections with mouse monoclonal anti-human CD20 (clone L26, Leica Microsystems, Tokyo, Japan) was performed on a fully automated BOND-MAX system (Leica Biosystems, Mount Waverley, VIC, Australia). The images of two or four independent cross-sections from one kidney were captured by a Nanozoomer S60 (Hamamatsu Photonics, Hamamatsu, Japan) and the degree of Daudi dissemination in the kidney was quantified using Image J.

### Statistics

The statistical significance was determined by Prism software (ver.7, GraphPad Prism, San Diego, CA).

## Results and Discussion

### Establishment of NOG-FcγR^-/-^ Mice

**Figure 1:**
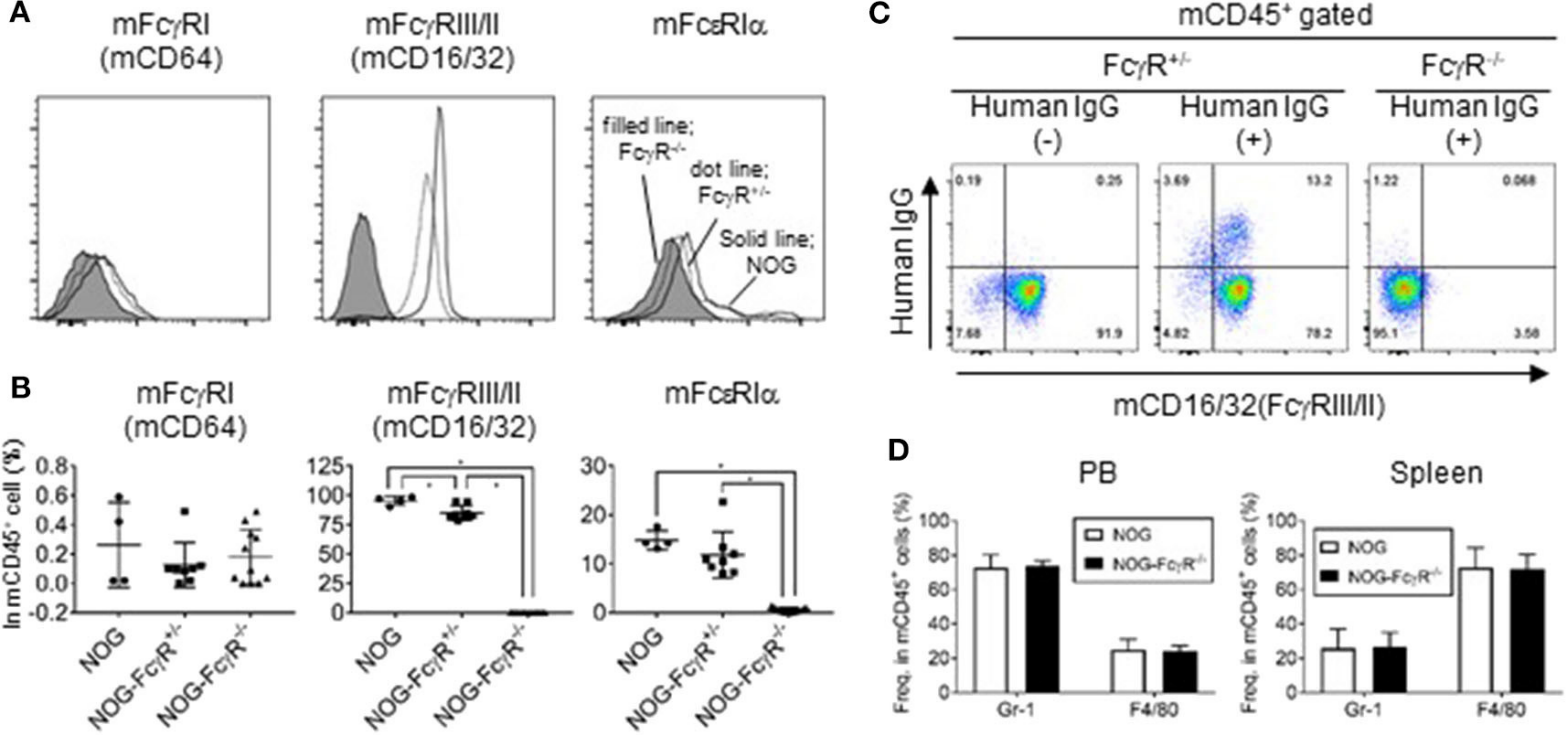
Absence of FcγR in leukocytes of NOG-FcγR^−/−^ mice. **(A)** Spleen cells from NOG, NOG-FcγR^+/−^, and NOG-FcγR^−/−^ mice were stained with the indicated antibodies and analyzed by flow cytometry. Representative FACS histograms for mouse FcγRI (CD64), FcγRIII/II (CD16/32), and FcεRIα. Solid line for NOG, dotted line for NOG-FcγR^+/−^ and filled histogram for NOG-FcγR^−/−^ mice. **(B)** The frequencies of FcR-positive cells among total mononuclear cells are plotted for NOG (*n* = 4), NOG-FcγR^+/−^ (*n* = 8), and NOG-FcγR^−/−^ mice (*n* = 11). **(C)** Failure of mouse monocytes from NOG-FcγR^−/−^ mice to capture human IgG. Trastuzumab (100 μg anti-HER-2 human IgG) was injected i.p. into NOG-FcγR^+/−^ and NOG-FcγR^−/−^ mice. Mononuclear cells prepared from peripheral blood (PB) 3 days after administration were stained with anti-human IgG, together with anti-mouse CD45 and CD16/32 antibodies for gating. Representative FACS plots are from three independent experiments. **(D)** Frequencies of mouse Gr-1^+^ neutrophils and F4/80^+^ macrophage/monocytes in peripheral blood (PB) and spleen. Means ± SDs are shown for NOG mice (*n* = 7 for PB and *n* = 4 for spleen) and NOG-FcγR^−/−^ mice (*n* = 7 for PB and *n* = 4 for spleen). Asterisks indicate the statistical significance by one-way ANOVA (*p < 0.001).

NOG mice and NOD-FcγR^−/−^ mice were crossed to introduce the defective mutations in both alleles of the *scid, IL-2rg, Fcer1g, and Fcgr2b* loci. FcεRIγ (FcR common γ) is the common subunit for FcγRI, III, and IV, and its deficiency leads to the absence of expression of FcγRI, III, and IV (23, 24). Due to the additional absence of FcγRIIB, NOG-FcγR ^−/−^ mice had no expression of any mouse FcγR. Indeed, flow cytometric analyses of spleen cells and PB confirmed the absence of mouse FcγRI (CD64), FcγRIII/II (CD16/32), and FcεRIα in the mononuclear cells of NOG-FcγR^−/−^ mice, while those present in NOG or NOG-FcγR^+/−^ mice (Figures 1A,B).

To establish that there was functional FcγR deficiency, we administered 100 μg Trastuzumab (anti-HER-2 antibody, human IgG1) into NOG-FcγR^+/−^ and NOG-FcγR^−/−^ mice by intraperitoneal injection. Analyses of PB 3 days after injection showed that a significant amount of human IgG was present on the surface of about 15% of mouse CD45^+^ cells in NOG-FcγR^+/−^ mice, and that these consisted of about 75% mouse neutrophils and 25% mouse macrophages/monocytes. In contrast, such human IgG was not detected in NOG-FcγR^−/−^ mice (Figure 1C), although the composition of mouse neutrophils and macrophages/monocytes was not different between the two strains (Figure 1D). This suggests that Trastuzumab was captured by FcγR on mouse phagocytic cells, including neutrophils and monocytes/macrophages.

We also measured the half-life of human IgG in NOG-FcγR^−/−^ mice, as a recent report demonstrated that Fc-FcγR interaction is a determinant for the rapid clearance of antibody in NOG or NSG (25). Doses (100 μg) of Rituximab (anti-CD20, human IgG1), Trastuzumab, or Mogamulizumab (anti-CCR4, human IgG1) were injected i.p. and levels of human IgG were monitored over 4 weeks. A plateau level of human IgG was maintained for 1 week, but rapidly decreased over the following week and became undetectable after about 2 weeks after injection. The rapid clearance of human IgG was not different between NOG-FcγR^+/−^ and NOG-FcγR^−/−^ mice. This result markedly contrasts with the long-term circulation of mouse IgG1 (MOPC-21) in PB, which persisted beyond 12 weeks (Supplementary Figure 2). Hence, our results demonstrate that mouse FcγR molecules are not involved in the pharmacokinetics of human IgG antibody. Another Fc receptor, neonatal FcR (FcRn), may be more relevant to the maintenance of human IgG proteins in mice (5, 26, 27).

### Antibody-Dependent Cytotoxic Effects of Innate Cells in NOG Mice

**Figure 2:**
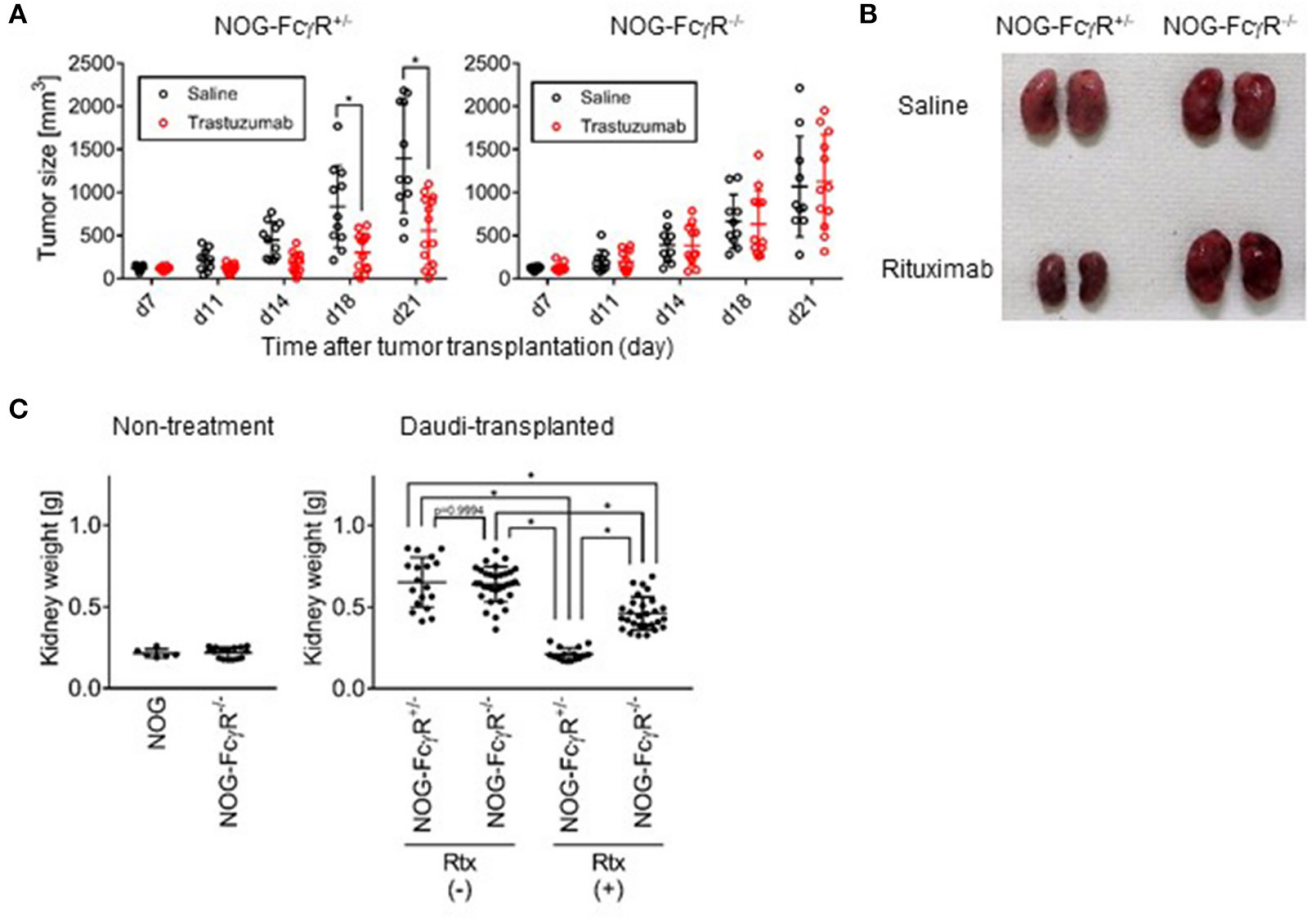
Abrogation of mouse innate cell mediated antibody dependent cytotoxicity in NOG-FcγR^−/−^ mice. **(A)** NOG-FcγR^+/−^ or NOG-FcγR^−/−^ mice were subcutaneously transplanted with a 2–3mm^3^ piece of 4–1ST tumor on day 0, and administered 50 μg Trastuzumab or saline by i.p. injection twice per week over 2 weeks starting from day 7. The tumor volume from individual mice is plotted with the average. NOG-FcγR^+/−^ (*n* = 11 or 14 for saline- or Trastuzumab-treated mice, respectively) or NOG-FcγR^−/−^ mice (*n* = 10 or 12 for saline- or Trastuzumab-treated, respectively); bars represent SD. Cumulative data from three independent experiments are shown. Asterisks indicate statistical significance by a repeated measure ANOVA with Sidak's multiple comparison test (*p < 0.0001, All the raw data, statistics, and plots of tumor sizes in each experiment are available in Supplementary Table 1). **(B)** NOG-FcγR^+/−^ or NOG-FcγR^−/−^ mice received 1.5 × 10^6^ Daudi cells intravenously, and 50 μg Rituximab or saline was injected i.p. on days 7, 14, and 21. The image shows representative kidneys resected from the indicated mice at day 23. **(C)** Plots of kidney weight from individual mice with mean ± SD for the indicated groups: normal NOG (*n* = 3), NOG-FcγR^−/−^ mice (*n* = 8), Daudi-engrafted NOG-FcγR^+/−^ (*n* = 9 or 8 for saline- or Rituximab-treated mice, respectively from two independent experiments), and Daudi-engrafted NOG-FcγR^−/−^ mice (*n* = 16 or 14 for saline- or Rituximab-treated mice from four or three independent experiments, respectively). Asterisks indicate statistical significance by two-way ANOVA with Sidak's multiple comparison test (*p < 0.0001, All the raw data and statistics are available in Supplementary Table 2).

Attempts to detect human NK-cell specific ADCC activity have often been confounded by regression of tumors after antibody treatment. For example, in our study, when a gastric cancer-derived HER-2^+^ cell line, 4–1ST, was inoculated into NOG mice and subsequently treated with Trastuzumab, tumor growth was strongly suppressed (Figure 2A). Interestingly, the inhibition of 4–1ST tumor growth was totally abrogated in NOG-FcγR^−/−^ mice (Figure 2A, Supplementary Table 1, Supplementary Figure 3). In another model, when we intravenously transplanted the CD20^+^ Daudi Burkitt lymphoma cell line in NOG- FcγR^+/−^ mice, Rituximab treatment alone strongly suppressed swelling in kidneys, where Daudi preferentially accumulates and forms colonies (Figures 2B,C). In contrast, the degree of the suppression of Daudi growth by Rituximab treatment in NOG-FcγR^−/−^ mice was reduced to about 30–40% of the level in NOG- FcγR^+/−^ mice (Figure 2C, Supplementary Table 2). This was also confirmed by immunohistochemistry using anti-CD20 antibody (data not shown). These results suggest that innate cells in mice, which include macrophages and neutrophils, actively killed human tumor cells by antibody-dependent mechanisms through the mouse FcγR molecules, and that NOG-FcγR^−/−^ mice were useful for eliminating these endogenous effects.

### Specific Detection of Human NK-Cell Mediated ADCC in NOG-FcγR^-/-^-hIL-15 Tg Mice

Previously, our group established NOG-hIL-2 Tg and NOG-hIL-15 Tg strains, which supported the development of human NK cells and the long-term persistence of human NK cells, respectively. These models are useful for examining the capacity of antibody drugs to activate human NK cells to induce ADCC. However, our results suggest that this ADCC involves two types of effector cells, human NK cells and mouse innate phagocytic cells. To segregate these two components, we generated NOG-FcγR^−/−^-hIL-15 Tg mice. The level of human IL-15 in NOG-FcγR^−/−^-hIL-15 Tg mice was comparable to that in NOG-hIL-15 Tg mice, and the period of persistence of human NK cells was not different between these strains (Supplementary Figure 4).

**Figure 3:**
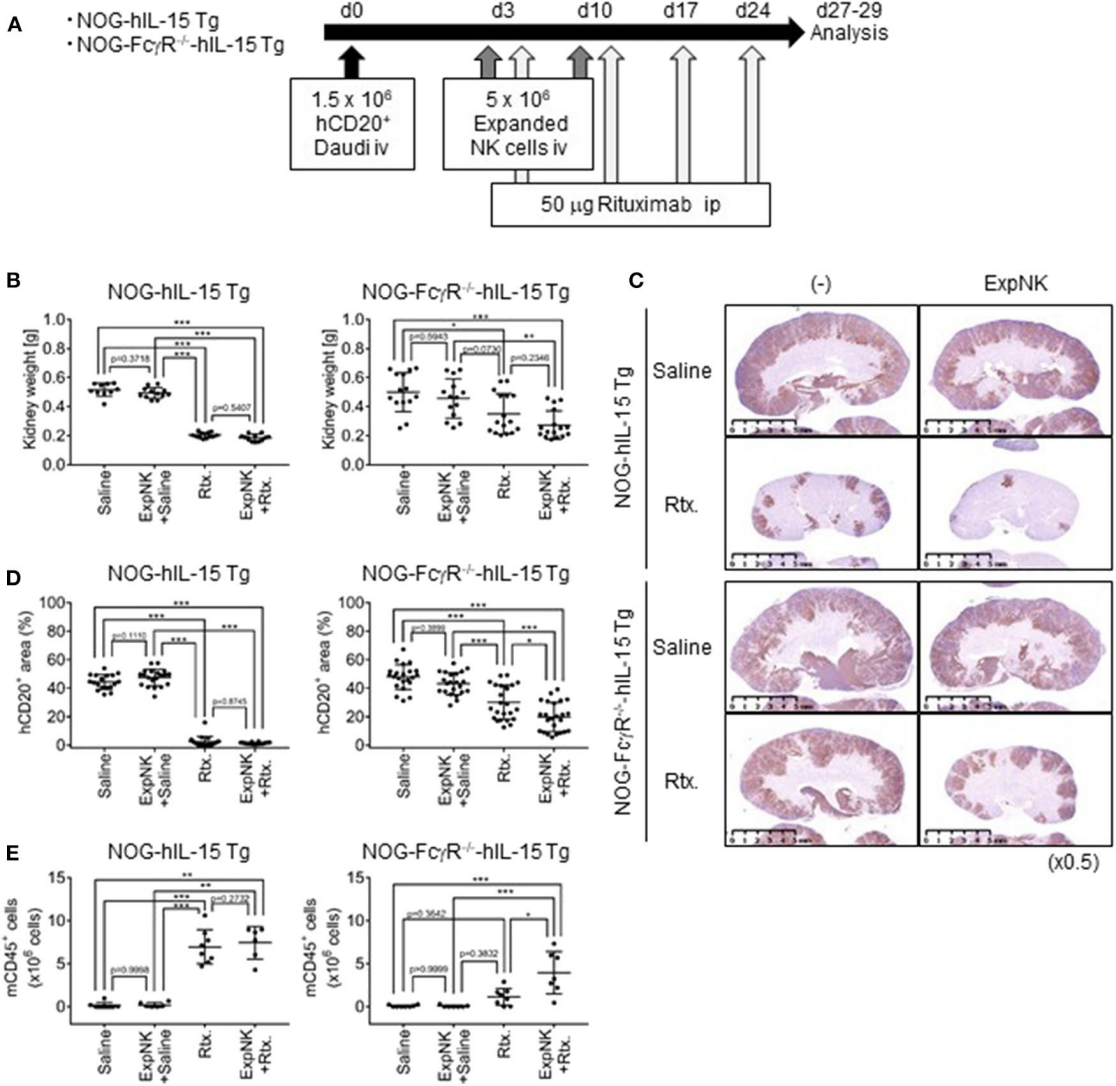
Specific detection of human NK cell-mediated ADCC in NOG-FcγR^−/−^-hIL-15 Tg mice. **(A)** Schema of experiments. NOG-hIL-15 Tg and NOG-FcγR^−/−^-hIL-15 Tg male mice received 1.5 × 10^6^ Daudi cells i.v., followed by 5 × 10^6^ *in vitro* expanded human NK cells at days 3 and 10. Rituximab (50 μg) was administered once per week over 3 weeks, starting at day 3; mice were analyzed at days 27 and 29. **(B)** Prevention of kidney swelling in NOG-FcγR^−/−^-hIL-15 Tg mice treated with combined therapy. Kidney weights from individual NOG-hIL-15 Tg (left) or NOG-FcγR^−/−^-hIL-15 Tg (right) mice are plotted with the mean ± SD in each mouse group. The NOG-hIL-15 Tg group consisted of five saline-treated, six NK-treated, six Rituximab-treated, six combined therapy-treated mice. For NOG-FcγR^−/−^-hIL-15 Tg, seven saline-treated, seven NK-treated, eight Rituximab-treated, eight combined therapy-treated mice. **(C)** Immunohistochemistry of kidneys from Daudi bearing mice in **(B)**. Tumors in kidney were detected using anti-CD20 antibody. Representative images are shown. **(D)** Quantification of Daudi dissemination by image analyses. **(E)** Prevention of BM destruction in NOG-FcγR^−/−^-hIL-15 Tg mice with combined therapy. The absolute numbers of mouse CD45^+^ cells in BM from individual mice are plotted with means and SDs. Cumulative results from two independent experiments are shown. Asterisks indicate the statistical significance by two-way ANOVA with Tukey's multiple comparisons test (*p < 0.005, **p < 0.001, ***p < 0.0001, All the raw data and statistics are available in Supplementary Tables 3, 4).

Next, we examined whether human NK-cell mediated ADCC was specifically detected in the new strain. Mice were inoculated with Daudi cells and subsequently subjected to three different therapeutic protocols: human NK cells, Rituximab, or a combination of the two (Figure 3A). When we used NOG or NOG-FcγR^−/−^ mice, transferred human NK cells were rapidly lost, and we could not detect protective effects in any of the mice (data not shown). When we compared NOG-hIL-15 Tg and NOG-FcγR^−/−^-hIL-15 Tg mice, transfer of human NK cells alone did not induce tumor suppression in either strain, as shown by increases of kidney weight (Figure 3B). As in NOG mice, Rituximab treatment strongly inhibited kidney swelling in NOG-hIL-15 Tg mice, and this inhibition was evident irrespective of the presence of human NK cells (Figure 3B, left). In contrast, in NOG-FcγR^−/−^-hIL-15 Tg mice, the combined therapy of human NK cells and Rituximab induced significant reductions in kidney weight compared to saline -treated or NK-cell transferred group. Rituximab-treated mice showed intermediate reduction in kidney weight and there was no statistical significance between this group and the combined therapy group (Figure 3B, right, Supplementary Table 3, Supplementary Figure 5). Immunohistochemical analyses also gave the consistent results (Figures 3C,D, Supplementary Table 4, Supplementary Figure 6). It was interesting, however, the image analysis of the multiple sections from immunohistochemistry demonstrated that the mice with combined therapy showed further enhanced suppression of Daudi when compared to the rituximab-treatment group, suggesting that human NK cells killed Daudi cells through ADCC (Figures 3C,D).

Bone marrow (BM) analyses also demonstrated that combined therapy was effective for preventing otherwise devastating BM destruction by Daudi cells. In NOG mice, Daudi cells cause a severe reduction of mouse CD45^+^ cells in BM by unknown mechanisms (data not shown). In NOG-hIL-15 Tg mice, Rituximab treatment rescued mouse CD45^+^ cells, irrespective of NK-cell transplantation, and mice without treatment or mice who received human NK cell transfer alone showed severe reductions in mouse CD45^+^ cells (Figure 3E). On the other hand, in NOG-FcγR^−/−^-hIL-15 Tg mice, only the group that received combined therapy avoided the destruction of BM (Figure 3E). These results suggest that specific detection of human NK cell-mediated ADCC is possible in this novel mouse strain.

Along with advances in antibody technology, the development of animal models providing “a proof of concept” for drug candidates has been urgently needed. In this study, we used Rituximab and Trastuzumab, which are specific for human CD20 and HER-2, respectively. We found that NOG-FcγR^−/−^ mice protected tumor cells from mouse innate cell-mediated cytotoxicity after antibody treatment. It is important to note that such cytotoxicity by innate cells in mice would affect various combinations of antibody and target cells, and that the degree of cytotoxicity is often difficult to predict for *in vivo* settings. For example, when we previously used an L428 cell line and anti-CCR4 antibody (Mogamulizumab), we found modest influences on cell growth (17). In addition, our previous report showed that HER-2-positive NCI-N87 was resistant to Trastuzumab treatment (18) in contrast to 4–1ST in this study. Furthermore, it was demonstrated that the growth of CD20^+^ Raji cells was not affected by Rituximab in human SIRPA and IL-15 knock-in (SRG-15) mice (16), while another group demonstrated that Raji cells were partially suppressed by Rituximab in NSG mice (28). Those differences in the biological consequences of treatments indicate that sensitivity to antibody drugs is partly determined by the features of immunological interactions between cancer cells and mouse innate cells. In the presence of antibody drugs, resistant cells like NCI-N87 and Raji may avoid mouse innate immunity by some mechanisms such as providing inhibitory signals, absence of stimulatory molecules, or expressing protective molecules, whereas susceptible tumor cell lines like 4–1ST or Daudi may strongly activate mouse innate cells through various molecules including FcγR. Interestingly, the degree of dependency on FcγR was different between 4–1ST and Daudi. The killing of 4–1ST was almost totally dependent on FcγR. In contrast, the partial suppression of Daudi by Rituximab in NOG-FcγR^−/−^-hIL-15 Tg mice suggests that some FcγR-independent killing mechanisms function in this combination. Although the amount of hIL-15 in NOG-hIL-15Tg mice was much higher than that of the level of endogenous mIL-15 (Supplementary Figure 7), it is not likely that the abundant hIL-15 activates such FcγR-independent mechanisms in mouse innate cells because of the absence of IL-2Rγ in NOG mice to transduce the cytokine signal. One possibility is complement-dependent cytotoxicity (CDC) (29, 30). Although activation of the complement system is incomplete in NOG mice due to a nonsense mutation in the C5 gene (31), other factors like the C3 fragments may mediate the partial elimination of Daudi cells. It was also suggested that the resistance of Raji cells to Rituximab was due to the modest level of CD20 and high levels of CD55 or CD59, both complement regulatory proteins (32). CD59, especially, might prevent the formation of C5-mediated membrane attack complex in SRG-15 mice. Disruption of the C3 or C1q genes in NOG-FcγR^−/−^-hIL-15 Tg mice will clarify the involvement of the complement pathway in the FcγR-independent mechanisms in the future and the development of NOG- FcγR^−/−^/C3^−/−^-, and NOG- FcγR^−/−^/C1q^−/−^-double deficient NOG mice is underway. Those models will be useful for evaluating ADCC in a wide variety of cancer cell lines and patient-derived cell lines (PDX).

Activation of mouse innate cells through FcγR may also be a problem in the development of novel forms of antibody with dual specificity, called bispecific antibodies (BsAbs). BsAbs include molecules with many types of architecture, with or without Fc portions (33), and are expected to have a broad range of functions, such as engaging two different cells or crosslinking different molecules on the surface of a single cell. When BsAbs have an Fc portion with binding capacity for mouse FcRs, the targeted cells could be affected by FcγR-expressing innate cells in mice, and validation of function could be compromised. It is rational to expect that NOG-FcγR^−/−^ mice could improve such situations.

A mouse strain that expresses human FcγRs and lacks all mouse FcγRs has been reported to be useful for antibody development (34, 35). In this strain, human IgG is captured by human FcγRs on mouse cells and activates pharmacodynamic pathways in a way similar to that in humans, although the effector cells are mice. Our NOG-FcγR^−/−^ mice should be able to provide another complementary model, when combined with NOG-hGM-CSF/IL-3 (36) or NOG-hIL-6 Tg mice (37). Because those models can induce development of human myeloid cells from human hematopoietic stem (HSC) cells, human HSC-transplanted NOG-FcγR^−/−^-hGM-CSF/IL-3 Tg or NOG-FcγR^−/−^-hIL-6 Tg mice would reconstitute a wide variety of human FcγR-expressing myeloid cells, together with human lymphocytes, in the absence of mouse FcγRs. The biological function of human IgG drugs could be better examined in these models than in mouse FcγR-competent models.

Finally, antibodies for human target molecules are still often produced in mice for research purposes. Such mouse IgG is easily captured by mouse FcγR molecules and activates mouse phagocytes. In this context, our NOG-FcγR^−/−^ mice would also be useful for small-scale pilot studies to obtain proofs of concept for academic research.

## Data Availability Statement

All datasets generated for this study are included in the article/Supplementary Material.

## Ethics Statement

The studies involving human participants were reviewed and approved by Research Ethics committee of CIEA. The patients/participants provided their written informed consent to participate in this study. The animal study was reviewed and approved by The Animal Experimentation Committee of CIEA.

## Author Contributions

IK and TT designed the project and completed the manuscript. IK and RI conducted experiments. KK performed the pathological examinations. All authors contributed to the article and approved the submitted version.

## Conflict of Interest

The authors declare that the research was conducted in the absence of any commercial or financial relationships that could be construed as a potential conflict of interest.
