# Supplementary material for: Improved Detection of in vivo Human NK Cell-Mediated Antibody-Dependent Cellular Cytotoxicity Using a Novel NOG-FcγR-Deficient Human IL-15 Transgenic Mouse
Source: Front Immunol. 2020 Oct 7;11:532684. doi: 10.3389/fimmu.2020.532684 (PMC7577188; doi:10.3389/fimmu.2020.532684)
Supplement: Supplementary file 1 [file Table_1.DOCX]

**Supplementary Materials**

Supplementary Table1. Raw data of tumor size and statistical analysis for Figure 2A

**
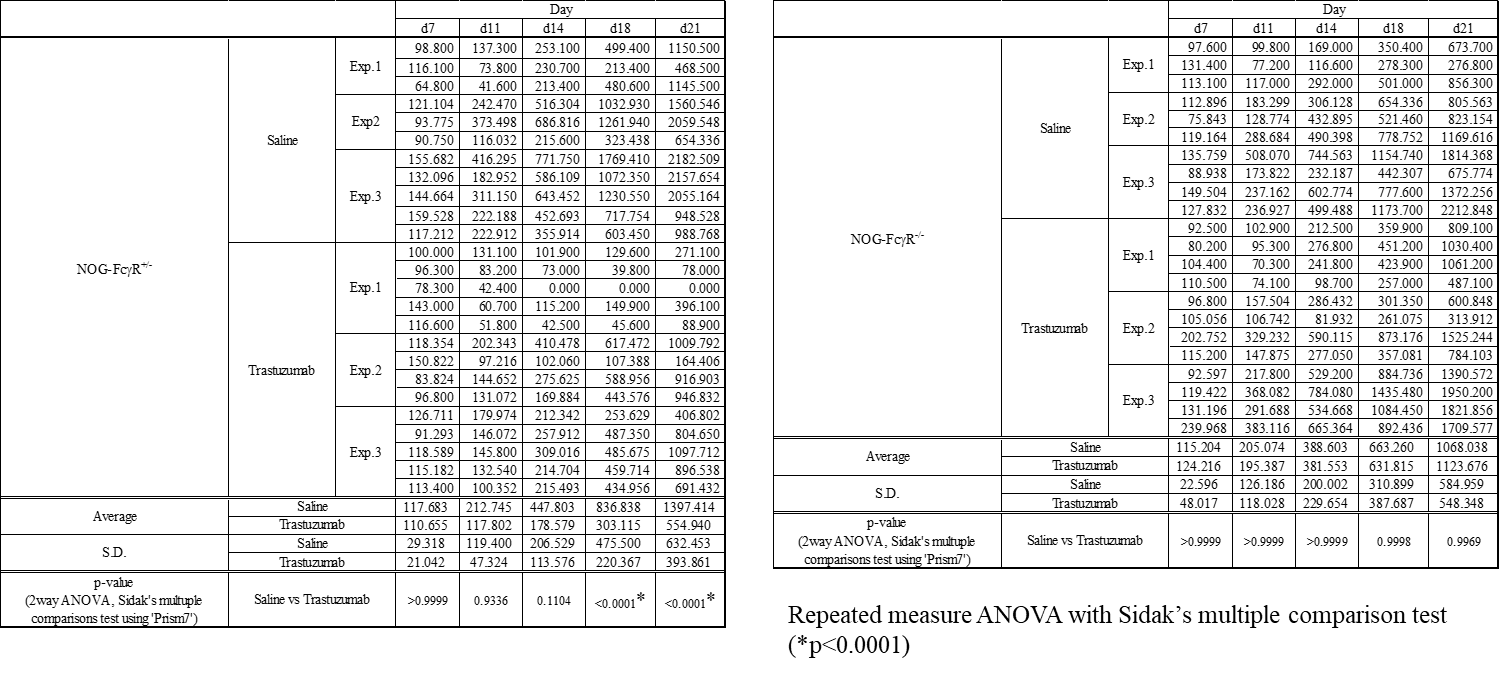
**

Supplementary Table2. Raw data of kidney weight and statistical analysis for Figure 2C

**
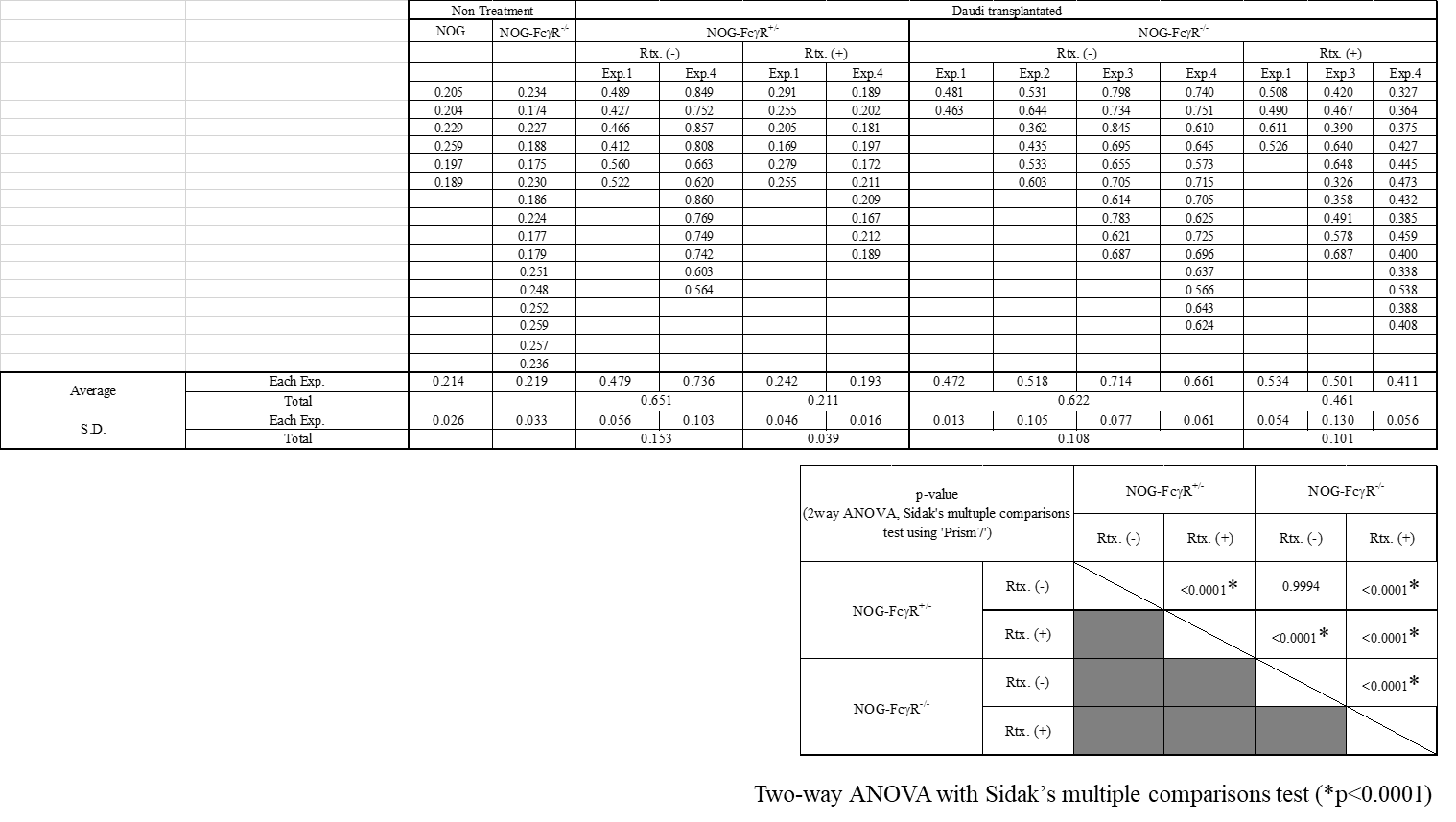
**

Supplementary Table 3. Raw data of kidney weight and statistical analysis for Figure 3B


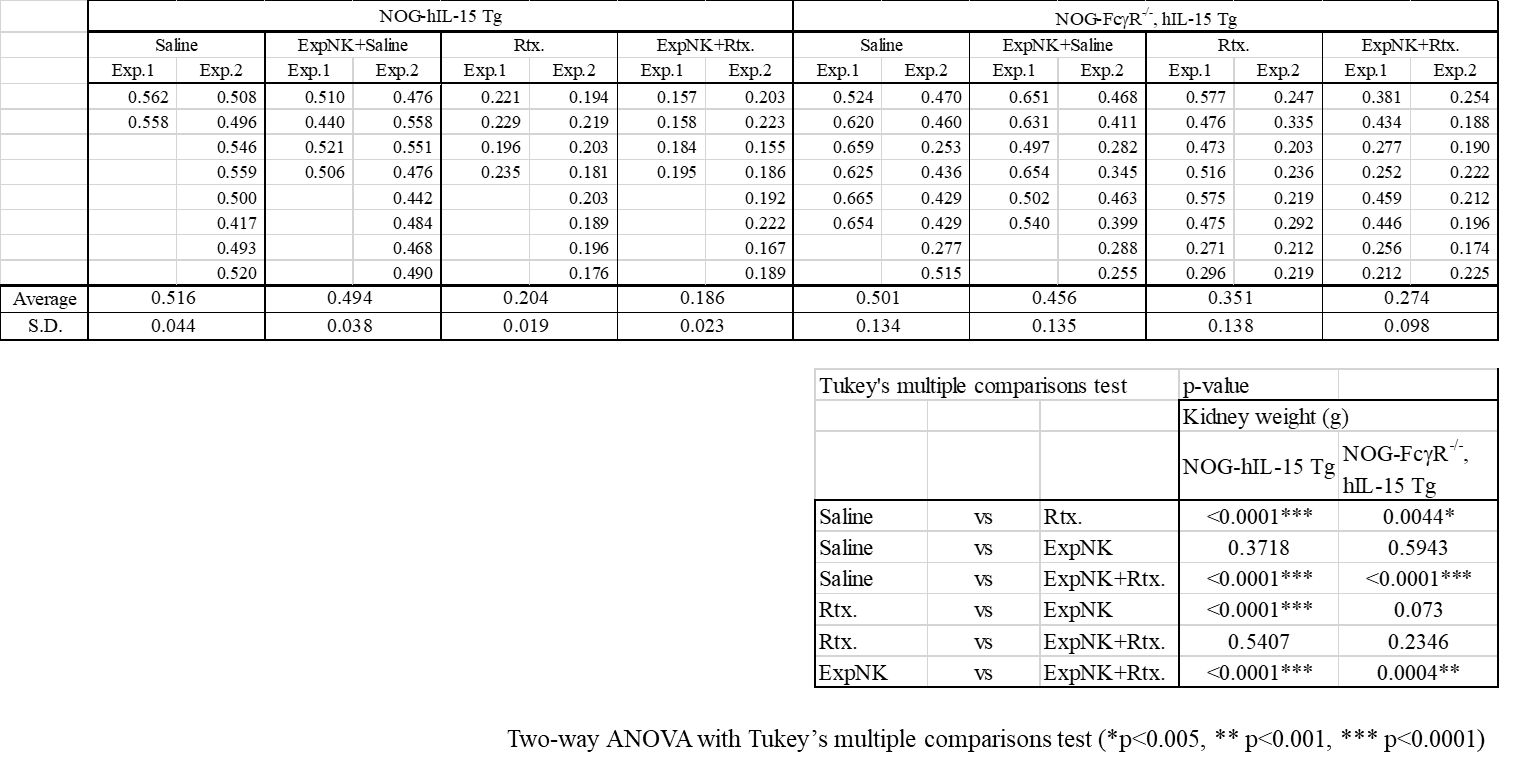


Supplementary Table 4. Raw data of tumor area and statistical analysis for Figure 3D

**
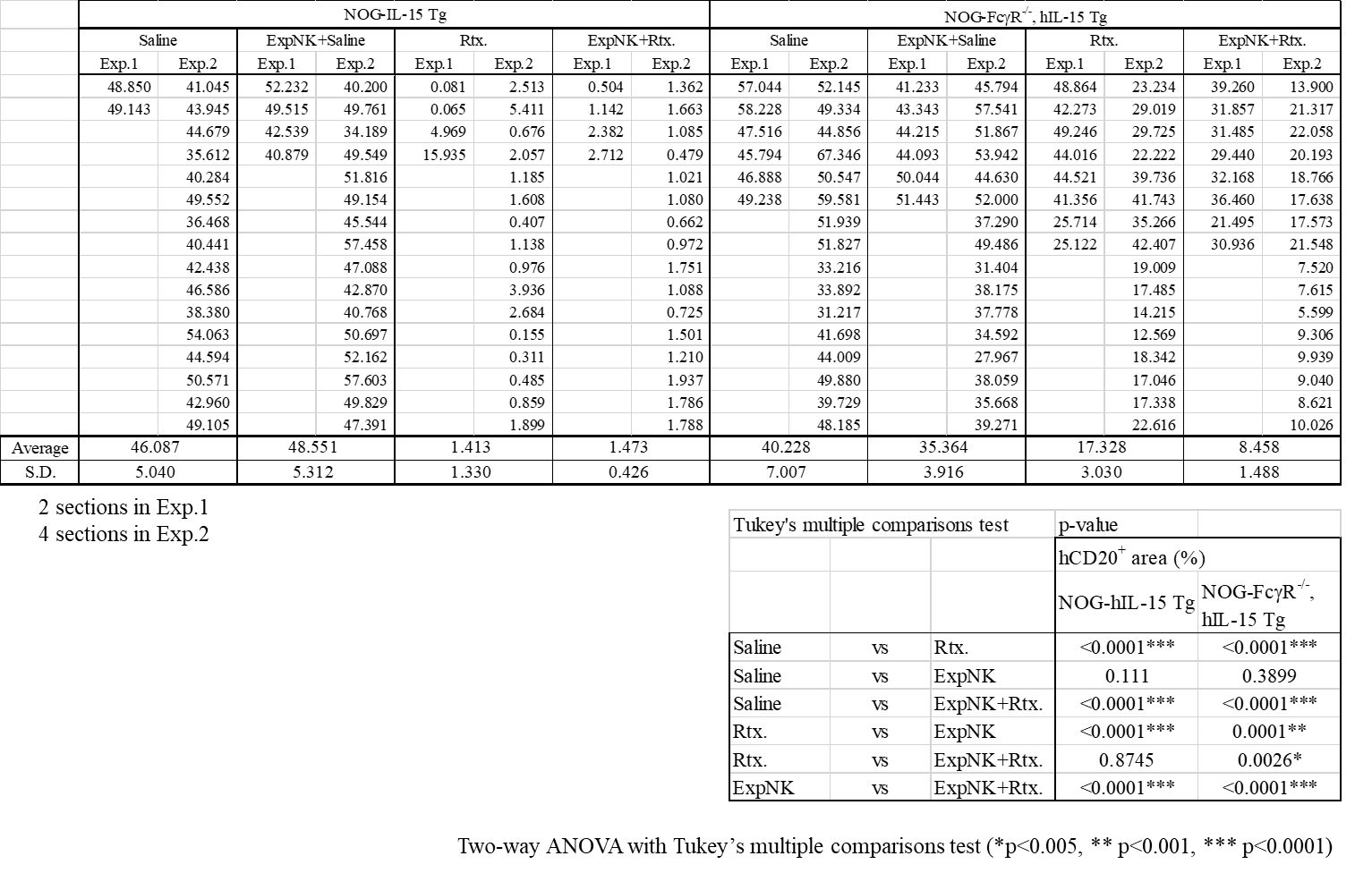
**

**
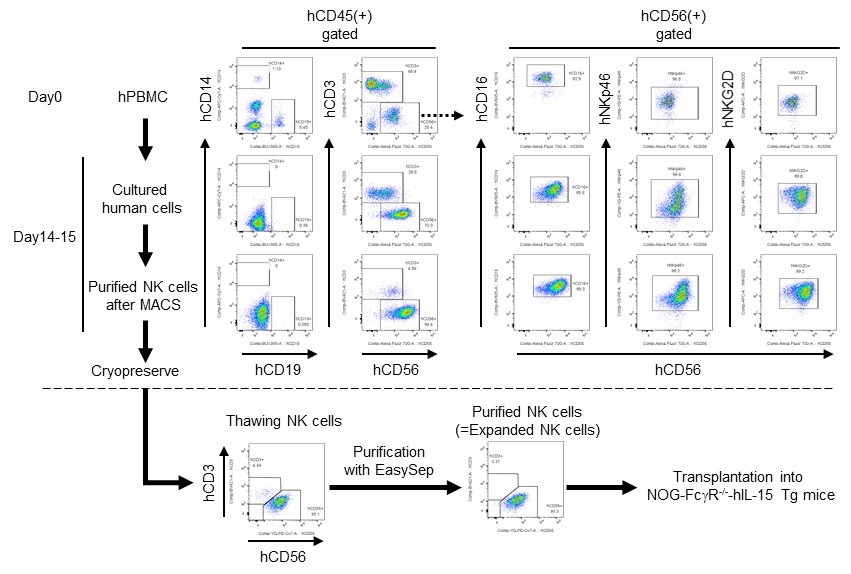
**

Supplementary Figure 1. In vitro expansion of human NK cells by BINKIT

Representative FACS plot of *in vitro* cultured human NK cells. Increased CD3^-^ CD56^+^ CD16^+^ cells were evident after *in vitro* culture, and those cells were subjected to MACS negative sorting. Before *in vivo* injection, cells were further purified using EasySep for depleting human T cells. The resulting purified human NK cell fraction was NKp46^+^ NKG2D^+^ and had a negligible number of CD3^+^ human T cells.


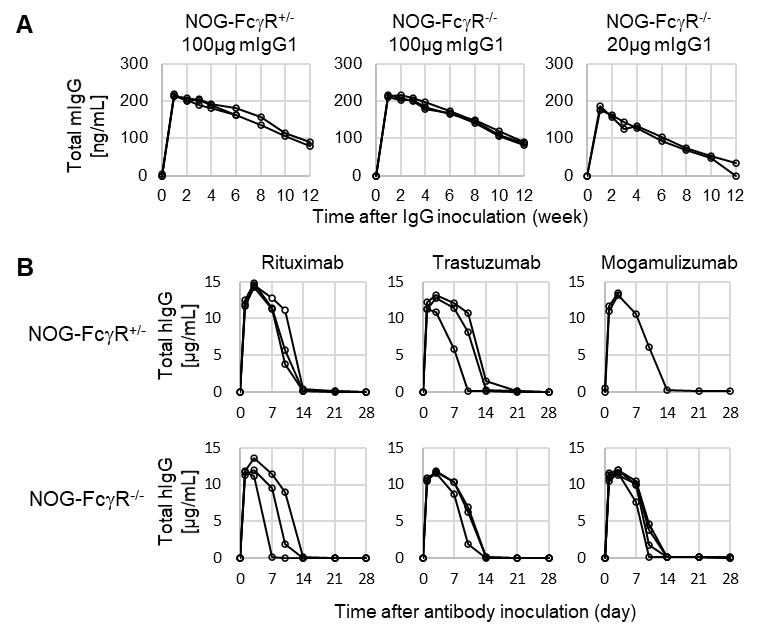


Supplementary Figure 2. Clearance of human IgG in NOG-FcγR^-/-^ mice.

Kinetics of human and mouse IgG in mouse plasma after injection of antibodies into mice.


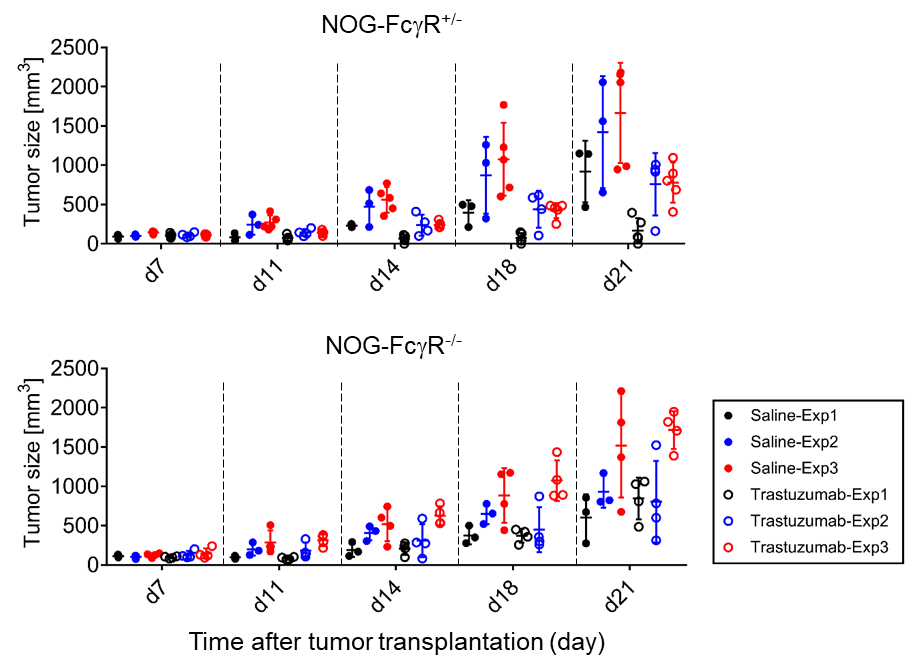


Supplementary Figure 3. 4-1ST growth in individual mice in independent experiments. Closed or open circles for saline- or Trastuzumab-treated mice, respectively. The same color represents the results from the same experiment set.


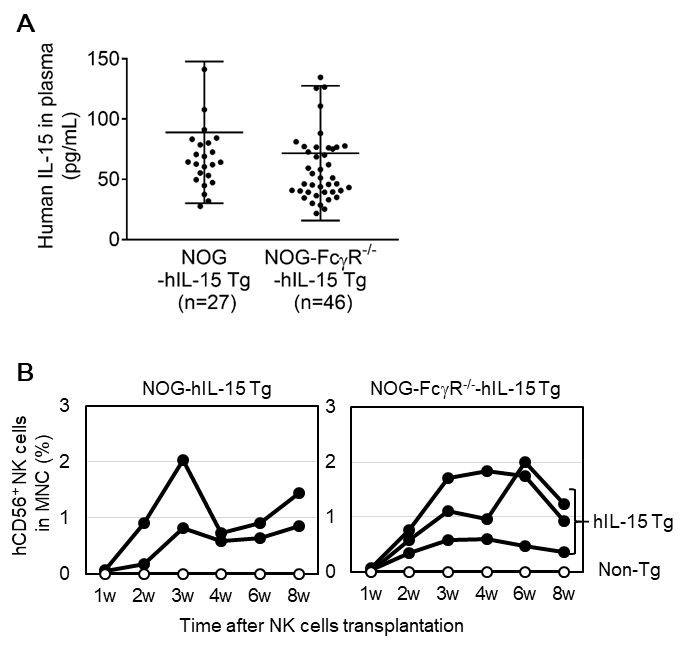

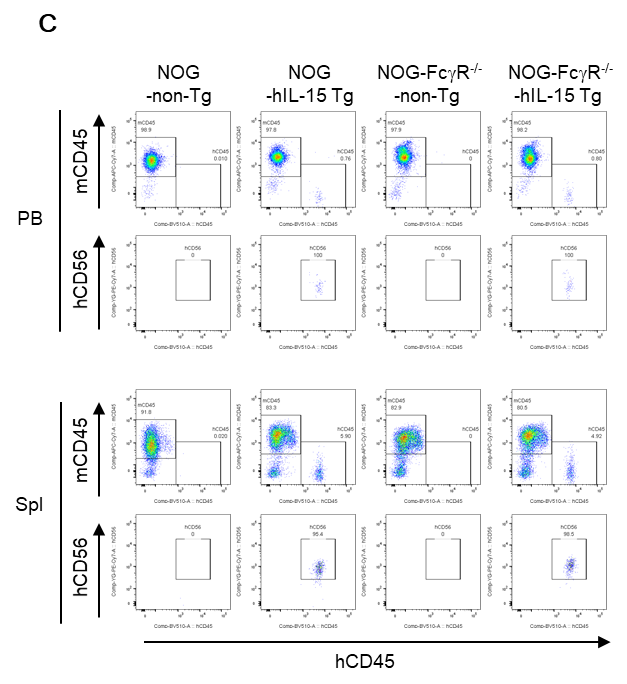


Supplementary Figure 4. Human IL-15 level in NOG-FcγR^-/-^-hIL-15 Tg mice and sustained engraftment of human NK cells.

(A) Plasma was collected from peripheral blood of NOG-hIL-15 Tg or NOG-FcγR^-/-^-hIL-15 Tg mice, and the level of human IL-15 was measured by using ELISA. (B) NOG, NOG-hIL-15 Tg, NOG-FcγR^-/-^, or NOG-FcγR^-/-^-hIL-15 Tg mice receiving 5 × 10^6^ *in vitro* cultured human NK cells. Cell presence was confirmed by flow cytometry (C).


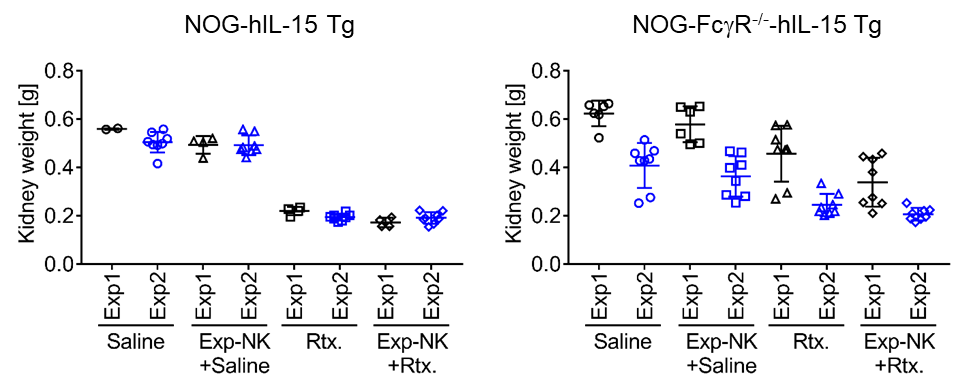


Supplementary Figure 5. Plots of kidney weight in individual mice from independent experiments


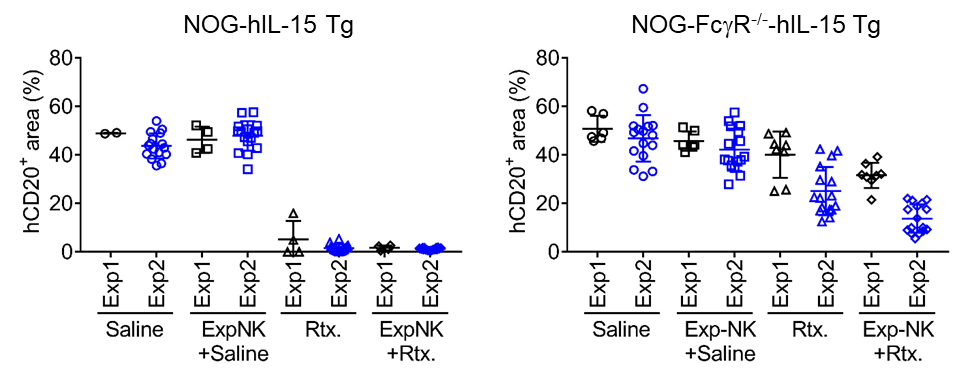


Supplementary Figure 6. Plots of tumor area in individual mice from independent experiments


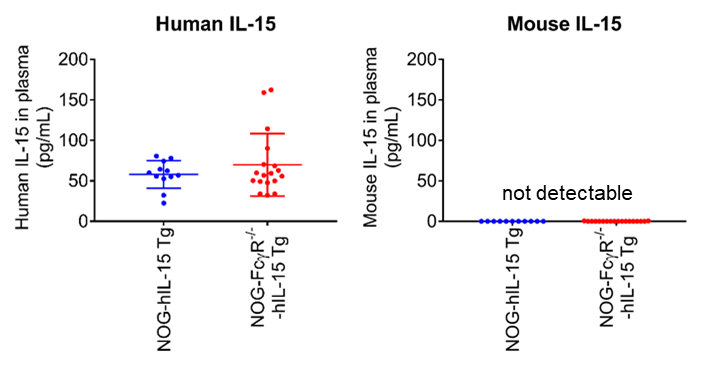


Supplementary Figure 7. Amounts of human IL-15 and mouse IL-15. Plasma was collected from peripheral blood of NOG-hIL-15 Tg and the levels of human and mouse IL-15 were measured by using ELISA.
